# Barriers to access to cancer care for patients from the conflict-affected region of the Nagorno-Karabakh Republic: A qualitative study

**DOI:** 10.1371/journal.pgph.0003243

**Published:** 2024-07-01

**Authors:** Alina Hovhannisyan, Celene Philip, Jemma Arakelyan, Gevorg Tamamyan, Stella Arakelyan

**Affiliations:** 1 Institute of Cancer and Crisis, Hematology Center After Prof. R. Yeolyan, Yerevan, Armenia; 2 Department of Chemistry, Hong Kong Special Administrative Region, City University of Hong Kong, Hong Kong, China; 3 Department of Public Health and Healthcare Organisation, Yerevan State Medical University after M. Heratsi, Yerevan, Armenia; 4 Paediatric Cancer and Blood Disorders Center, Hematology Center after Prof. Yeolyan, Yerevan, Armenia; 5 Usher Institute, Advanced Care Research Centre, University of Edinburgh, Edinburgh, United Kingdom; University of Toronto, CANADA

## Abstract

Access to essential health services is a basic human right, yet many cancer patients living in conflict-affected regions face multiple obstacles to service use. The (former) Nagorno-Karabakh Republic was a conflict-affected region in the South Caucasus populated predominantly by ethnic Armenians. Multiple acute armed conflicts, the recent military occupation of the region, and the prolonged military blockade of the Lachin Corridor (a humanitarian corridor connecting Nagorno-Karabakh to Armenia) exacerbated existing social, health, economic, and political fragilities in this region. As a result, cancer services were disrupted, with limited clarity on how the ongoing military blockade of a humanitarian corridor affected cancer patients’ experiences of accessing cancer care locally and in bordering Armenia. Our study aimed to describe the experiences of patients from Nagorno-Karabakh in accessing the cancer care services they needed. We conducted remote semi-structured interviews with adult (aged ≥18 years) cancer patients receiving cancer care from three university hospitals in Armenia and face-to-face interviews with cancer care professionals from these hospitals. Interviews were conducted during the blockade of the Lachin Corridor between March and May 2023. Data were analysed thematically using a deductive approach. Twelve adult cancer patients (9 women) and 12 cancer care professionals participated. A key barrier to accessing cancer services was attributed to the Azerbaijani military occupation of the region and the blockade of a major roadway connecting Nagorno-Karabakh to Armenia. Patients talked in length about the challenges of finding transport and travelling long distances to reach essential cancer services in Armenia. Policies of free anti-cancer medication provision and decentralised medication supply were paused because of the military occupation, affecting patients’ timely access to anti-cancer medication. Out-of-pocket expenses for treatment, anti-cancer medication, travel, and temporary accommodation in Armenia placed a significant financial burden on cancer patients, exacerbated by the humanitarian crisis. Conflict-affected regions blockaded by military forces lack the capacity and targeted support to sustain their essential health services and provide care to those in need of life-saving treatments. Coordinated action from national and international organisations and governments is urgently needed to enhance humanitarian assistance and healthcare support to patients, their families and wider communities affected by military blockades and armed conflicts.

## Introduction

Global estimates suggest that over 19.3 million people were diagnosed with cancer and 10 million died from cancer in 2020, making it one of the leading causes of global morbidity and mortality [1]. Cancer incidence is lower in low- and middle-income countries (LMICs); however, cancer-related mortality is significantly higher compared to the rest of the world [2]. Cancer care provision is a pressing issue in LMICs, particularly in those that are affected by armed conflicts and/or military blockades. Mostconflict-affected countries are among the least developed and poorest in the world [3]. Nearly a quarter of the global population lives in these fragile contexts, which are commonly characterised by the combination of exposure to multiple risks and insufficient coping abilities to manage, absorb or mitigate those risks at the community, systems, and country levels [3]. In conflict-affected countries, fragile health systems struggle to deliver essential health services and cater to population healthcare needs. As an example, recent armed conflicts in Syria, Ukraine, Iraq, Yemen, Sudan and Myanmar adversely affected countries’ healthcare infrastructure, resulting in limited availability of healthcare facilities, medical equipment and therapeutics and a lack of specialised healthcare professionals to meet care demands of vulnerable populations [4–9]. Cancer prevalence and mortality are expected to rise significantly in these countries due to the lack of adequately functioning cancer diagnostic and treatment services and the shift in funding towards military and acute care services [4]. This is supported by recent review findings suggesting positive associations between armed conflicts and increases in the incidence and mortality of breast and cervical cancers [10].

This paper reports on barriers to accessing cancer care in the humanitarian crisis for patients from the Nagorno-Karabakh Republic (also known as the Republic of Artsakh), which ceased to exist at the time of the paper’s publication due to the escalation of the regional armed conflict. The Nagorno-Karabakh Republic was a conflict-affected region in the South Caucasus, lying between the Armenia-Azerbaijan border. Historically, it was a partly self-governing region of the Soviet Union populated predominantly by ethnic Armenians and some surrounding districts by Azerbaijanis [11,12]. After the collapse of the Soviet Union in 1991, Nagorno-Karabakh was a de facto independent state. Its political status gained traction and stayed unresolved since its self-declaration of independence from Azerbaijan in 1994. September 2020 witnessed an outburst of fighting, resulting in Azerbaijan taking control of one-third of Nagorno-Karabakh. This conflict ended in November 2020, with *‘temporary peace’* negotiations brokered by Russia [11,12]. Nagorno-Karabakh became an isolated state connected with Armenia by a Russian-controlled humanitarian corridor known as the Lachin Corridor. On 12 December 2022, the Lachin Corridor was blockaded by Azerbaijani troops, exacerbating existing social, health, economic, and political fragilities in the region [12]. The movement of people and goods (food, fuel, medicines and medical supplies) across the Lachin corridor became constrained, endangering people’s lives. Access to essential healthcare was a major challenge in the region, limiting and, in some cases, completely disrupting the availability of services for vulnerable and at-risk population groups including, children, women and the elderly. Furthermore, all cancer prevention and care programs were paused, compromising access to early detection and life-saving treatments [12,13]. The information on cancer patients’ lived experiences in this climate of prolonged humanitarian crisis remained limited. We, therefore, conducted this study to describe the experiences of cancer patients from the Nagorno-Karabakh Republic in accessing cancer care services both locally and in bordering Armenia.

## Materials and methods

This was a qualitative interview study exploring barriers to accessing cancer care services in patients from the Nagorno-Karabakh Republic during the military blockade of the Lachin corridor from the perspectives of cancer patients and cancer care professionals.

### Study context

The Nagorno-Karabakh Republic was a contested mountainous region (1700 square miles) located between Armenia and Azerbaijan and populated by approximately 120,000 ethnic Armenians, including 30,000 children. Limited data exists on cancer incidence and prevalence rates, stage at diagnosis and treatment outcomes in ethnic Armenians of the Nagorno-Karabakh Republic; we, however, expect a similar incidence rate to that among adults in Armenia, i.e., crude cancer incidence rate of 266.9 per 100,000 people [14]. Before the military occupation of the region by Azerbaijani troops in 2020, cancer care in the region was mainly provided by the Oncology Centre in the capital city of Stepanakert, contracted by the Ministry of Health of Nagorno-Karabakh. At the time, essential cancer services were publicly co-funded, while for comprehensive cancer care, including radiation therapy and surgery, cancer patients had to access services in Armenia. Since 2019, radiation therapy and surgical treatment have been provided free of charge for all cancer patients in Armenia. However, demand for these services exceeds supply, forcing many cancer patients have to co-pay for cancer treatment [15].

### Participants and recruitment

The study participants were cancer patients and cancer care professionals. Eligible patients were adults (aged ≥18 years) at the time living in Nagorno-Karabakh who had a confirmed (and disclosed) diagnosis of cancer and received cancer care from the three hospital study sites in Armenia. In the Armenian cancer care context, disclosure of cancer diagnosis to patients is a contested and sensitive issue and subject to primary caregivers and family consent. Eligible cancer care professionals were clinical oncologists, clinical oncology residents or nurses who provided cancer care for patients from Nagorno-Karabakh. The study sites were three main university hospitals providing cancer services in Armenia: The National Oncology Centre, the Haematology Centre, and the Mikaelyan Institute of Surgery. Cancer services provision in the Haematology Centre is more specific to blood and solid tumours, whereas the Mikaelyan Institute of Surgery provides surgical treatment and chemotherapy for patients with solid tumours. The National Oncology Centre is the only centre in Armenia offering comprehensive cancer care services that combine specialist surgery integrated with chemotherapy, radiotherapy and haematology services as well as palliative care and psychosocial support.

The recruitment of the participants was carried out through a convenience sampling method. Three study site champions (clinical oncologists) were recruited through the team’s professional networks to support the convenience sampling of participants, eight (four patients and four cancer care professionals) from each study site. Potentially eligible patients were initially contacted by the site champions via phone to inform them about the study. For patients who agreed to take part in the study and their contact details to be shared, a member of our team initiated a follow-up phone call to explain the study. The information sheet was read to patients to aid decision-making and oral informed consent was sought and documented prior to the remote interview being conducted. Of the 14 patients approached, two refused to partake in the study without giving any reason. To build rapport between the interviewer and cancer patients, remote interviews took place over video calls. One patient from Nagorno-Karabakh was in a hospital site for an extended period due to a lack of housing in Yerevan (the capital of Armenia); this patient provided written consent and opted for the face-to-face interview. Potentially eligible cancer care professionals were approached by the site champions to introduce the study and provide information sheets. All potentially eligible cancer care professionals approached agreed to take part in the study and for study champions to share their contacts with the research team. Cancer care professionals provided written consent and opted for face-to-face interviews.

### Data collection and analysis

Data were collected during the ongoing blockade of the Lachin Corridor between 16 March and 4 May 2023. The individual semi-structured interviews were conducted by two female researchers (AH and CP) with training in public health and qualitative research, as applied to the study of health services research. Healthcare professional interviews were conducted prior to patient interviews. In developing the interview topic guide, we draw on Levesque et al. [16] conceptualisation of access to healthcare services. The topic guide focused broadly on five dimensions of accessibility of health services (approachability, acceptability, availability and accommodation, affordability, and appropriateness) and the ability of patients to interact with these dimensions to generate access (ability to perceive, ability to seek, ability to reach, ability to pay, and ability to engage). The topic guides are provided in S1 and S2 Tables.

Cancer care professionals were asked open-ended questions about service availability and accessibility for patients from Nagorno-Karabakh. Patient participants were asked open-ended questions relating to their experiences of accessing cancer services locally in Nagorno-Karabakh and Armenia. Patient and care professional self-reported socio-demographic information (age, marital status, education level, employment status, and geographical residence urban vs rural) was also collected. The format was relatively flexible, with the technical aspects (i.e., welcome and introduction, a brief presentation of the research, recording, etc.) being covered before the actual interview. The interviews lasted between 25–45 minutes and were audio-recorded using an encrypted recorder. The recordings were saved on a password-protected computer.

Audio recordings were transcribed verbatim by a single member (AH) of the research team. Transcripts were anonymised with an assigned study number. A deductive thematic analysis approach was used [17]. Two researchers (AH and CP) read transcripts several times to familiarise themselves with the data and establish a general thematic tone. A sample of transcripts from the first few interviews was coded by AH and CP, integrating Levesque et al. [16] five dimensions of accessibility of health services with new codes emerging from data. The initial coding framework was peer-debriefed with GT and SA. Following discussions, the research team reached a consensus which led to the formulation of a final coding framework that was deductively applied to all the transcripts. The data from patients and cancer care professionals were coded separately, but the analysis was undertaken conjointly. We identified recurring patterns in the data leading to the development of themes. Themes were evaluated critically to identify emerging patterns and associations.

### Ethics statement

Ethical approval was sought from the Ethics Committee of the Yerevan State Medical University [REF N6-3/2023]. Informed written consent for participation in the study was sought from the general manager of each study site prior to data collection. On entry to each study site, the researchers greeted staff in targeted departments and had a short information session with staff. All questions about the study aim and how data will be handled were addressed during the information session. Individual written informed consent was obtained from cancer care professionals, and oral informed consent was sought from cancer patients (except one patient who provided written consent) before interviews. Oral informed consent to participation was documented by the researcher on the patient consent forms before conducting the actual interviews. Assurance of anonymity and confidentiality were provided to both professionals and patients. Assurance was also given regarding the freedom to withdraw from the study at any time without any negative consequences.

## Results

A total of 24 participants (12 cancer patients and 12 cancer care professionals) were interviewed. The majority of cancer patients were married women (with a mean age of 50 years), diagnosed with cancer within five months up to two years prior to the interview. Cancer care professionals were predominantly women, clinical oncologists with a range of 3–18 years (with a mean of 6.6 years) of clinical experience. Participant characteristics are presented in Table 1.

**Table 1:** Participant characteristics.

Characteristics	Cancer patients	Healthcare professionals
**Age** (y, mean, range)	50y, 36-73y	31y, 26-52y
**Gender**		
Woman	9	11
Man	3	1
**Area residence**		
Urban	9	12
Rural	3	-
**Employment status**		
Full-time employed	3	12
Unemployed	9	-
**Educational attainment**		
Vocational education	5	-
Higher education	7	12
**Clinical setting**		
Haematology Centre	4	4
National Oncology Centre	5	4
Mikaelyan Institute of Surgery	3	4
**Clinical role**		
Clinical oncology resident	-	4
Clinical oncologist	-	8
**Clinical experience** (y, mean, range)	-	6.6y, 3-18y

Qualitative analysis of the patient and care provider interviews revealed several key factors impacting patients’ access to cancer services across the five service level dimensions and the corresponding patient-level abilities of the Levesque conceptual framework of access to health care. These barriers are summarised in Table 2.

**Table 2:** Barriers facing patients in accessing cancer care across the five dimensions of accessibility.

Dimensions	Barriers
Approachability	Blockade of Lachine corridor connecting Nagorno-Karabakh to Armenia Seeking out support with evacuation from the Red Cross Logistical challenges to support timely evacuation Proactive brokering from healthcare professionals
Availability and accommodation	Long-distance travel to reach essential services in Armenia Difficulties accessing transport Lack of local cancer services Lack of anti-cancer medication Dislocation from home while accessing treatment Dislocation from social support Inappropriate and/or unacceptable accommodation
Acceptability	Low interpersonal trust in local services Low institutional trust in local services Scarcity of local cancer care providers
Appropriateness	Quality of local services
Affordability	Out-of-pocket expenses for cancer treatment Out-of-pocket expenses for anti-cancer medication ‘Hidden costs’ associated with travel and accommodation Lack of financial protection

### Approachability of cancer services

A key barrier to timely access to cancer services was attributed to the Azerbaijani military occupation of the Nagorno-Karabakh Republic and the blockade of major roadways that connected this region with Armenia. Cancer patients were very vocal about their struggles to negotiate access to the Lachin corridor to reach services, with some patients missing their routine cancer surveillance as a result. Patients were avoiding travel to Armenia unless postponing treatment posed a critical threat to their lives. The ongoing blockade of the roadways meant that cancer patients had to seek out external support from humanitarian organisations like the Red Cross as one health professional put it *‘to negotiate their secure transfer’* to Armenia. Coordinating travel from Nagorno-Karabakh was a time-consuming and complex process, but also unsafe, which resulted in some patients delaying their treatment:

*“I used to come to Armenia for routine examinations every three months, however, I have missed my recent appointments because of the blockade of roads [to Armenia]. Now I have decided to turn to the Red Cross for help to reach the cancer services I need.” [Cancer patient, man]*

*“I have a patient who was supposed to receive the next course of chemotherapy, but he couldn’t travel to Armenia because of the blockade. There was no way to transfer him here, so the treatment was postponed. It was challenging before, and it is doubly challenging now.” [Oncologist, woman]*

Cancer care professionals talked about a decreased patient flow from Nagorno-Karabakh attributed to the blockade of the Lachin corridor and the logistical challenges the Red Cross staff faced in supporting the emergency and medical evacuation of patients. Proactive brokering was essential to speed up patients’ evacuation, increasing pressure and time demands on cancer care professionals.

*“The main issue right now is transportation. I doubt that all those in need of cancer care will be able to reach us. There is also a capacity issue, and it is possible that the Red Cross will not be able to keep up with the demand in the future. When they [Azerbaijani troops] first blockaded the roads, it took the Red Cross 1.5 months to determine a process of secure transfer of patients to and from Armenia. This also affects the quality of services we provide, including delays in treatment and, therefore, patient prognosis for survival.” [Oncologist, woman]*

*“Provision of cancer care in the context of blockade is challenging. We need to justify each request for medical evacuation. Patients will be denied free and safe access through the Lachin corridor if they simply show up at the checkpoint and show their medical documentation to Azerbaijani soldiers.” [Oncologist, woman]*

The complexity of processes patients had to undergo to qualify for support from the Red Cross prompted some patients to find alternative pathways to staying connected with their care providers, including phone calls to private cell phones:

*“The blockade [the Lachin corridor] is the only difficulty for now. I frequently contact my doctors via Viber and WhatsApp, and they [health professionals] always answer my calls”. [Cancer patient, man]*

### Availability and accommodation of cancer services

Patients talked in length about the challenges of finding transport and travelling long distances to reach essential cancer services in Armenia. All patients preferred to receive cancer care locally to avoid repeated crossing of the checkpoint but had to travel to Armenia because limited cancer services were on offer in Artsakh. Patients received the necessary treatment and drug supplies along with any at-home care instructions in Armenia and continued their treatment in Artsakh if their condition allowed it:

*“It is difficult for them [patients] to travel back and forth all the time. Some patients require repeated weekly tests, and if we notice a change in their tests, they need to return for further examinations. The treatment burden is enormous for these patients.” [Oncologist, woman]*

Cancer care professionals noted that long-distance travel was especially an issue for patients at risk of rapid decompensation, those who needed palliative care, and patients with major comorbidities (eg, cardiovascular diseases, diabetes). One care professional described how treatment had to be delayed because of the patient’s health deterioration during the actual journey to Armenia:

*“There are many cancer patients who have comorbid conditions, such as heart problems and high blood pressure. During the stressful and potentially dangerous travel to Armenia, they are at an increased risk of rapid health deterioration. I had instances where patients called and asked to reschedule the appointment as they felt unable to carry on with their journeys” [Oncologist, woman]*

Further, the military occupation of central roads presented a significant challenge to the movement of essential medical supplies, food and fuel. One patient described how they were forced to receive treatment in Armenia as they struggled to find a *‘trusted mediator’* to cross the Azerbaijani checkpoints with cancer medications they needed:

*“I must get my chemotherapy treatment in Armenia because there are no cancer medications here in Artsakh. Some drugs I need must be kept in the refrigerator at a certain temperature. So, I ask myself: Who can I trust to transport my medicines? How can I be confident they would follow all storing and handling recommendations?” [Cancer patient, woman]*

Among many challenges experienced by cancer patients, poor access to safe and secure housing in Armenia presented an additional and significant issue. Patients talked about poor living arrangements in temporary housing accommodations (hostels) or emergency shelters provided by the Armenian Government. To ease access to treatment, some patients were renting privately or sheltering with extended family or friends, leaving behind their families and children.

*“Hostels are really bad in Armenia. They are in very poor conditions, but where we can go.” [Cancer patient, man]*

*“Well, during my treatment in Armenia, I am away from my family and children, staying at a friend’s house. It’s very difficult when you do not have shelter. I am staying with my friend but this is out of my comfort zone.” [Cancer patient, woman]*

Cancer care professionals expressed frustration with a lack of material support for patients from Nagorno-Karabakh. Housing in hospitals was often considered as a last resort if other options were exhausted by the patients:

*“There should be a dedicated space for these patients, not some hostel with lacking sanitation and proper living conditions. There should be a tailored space where patients can both live comfortably and receive the treatment they need.” [Clinical resident, woman]*

*“Many people come to the hospital with no place to stay. The Armenian Government provides accommodation for a maximum of two days. For example, there was a patient in our ward with no place to go, and we had to let them stay in the hospital as it was unsafe to let them go.” [Clinical resident, woman]*

### Acceptability and appropriateness of cancer services

Almost all cancer patients emphasised that Armenian hospitals provide higher quality care and are far more technologically advanced in comparison to local services. A lack of local cancer care providers and services was noted. Health professionals in Armenia were described as more person-centred and professional in their approach to care, and commitment from care professionals to better supporting cancer patients from Nagorno-Karabakh was noted. Higher levels of institutional and interpersonal trust prompted patients to travel to Armenia despite obstacles caused by the blockade:

*“There is a huge difference between healthcare in Armenia and Artsakh. I prefer to receive treatment in Armenia because the quality of care is much better. As soon as the roads re-open, I will go back to continue the treatment in Armenia as we lack cancer doctors here and they are lagging behind.” [Cancer patient, man]*

*“I was examined in Artsakh several times but they were not able to put the correct diagnosis. I was diagnosed with cancer in Armenia”. [Cancer patient, woman]*

*“Of course, patients from Artsakh seek care in Armenia as they commonly have low trust in the quality of treatment they receive back home.” [Oncologist, man]*

A health professional spoke about patients’ frequent calls in distress when seeking care locally in Artsakh. Cancer services were described as inappropriate for meeting the complex care needs of cancer patients. Patients frequently developed side effects from their treatment and sought remote support from professionals in Armenia:

*“One of the problems that I often see is developing complications or side effects from cancer treatment administered in Artsakh. We then have to immediately establish contact with patients and care providers by phone to support them remotely. However, it is very hard to fully address the complications or provide targeted support without proper face-to-face supervision of these patients. [Clinical resident, woman]*

Some patients and professionals also talked about the impact of the military occupation of Nagorno-Karabakh and the prolonged blockade on patients’ psychosocial well-being and the need for providing psychosocial support services. This is in part because instability on the borders was a cause of patient distress and anxiety, especially for patients whose family members joined the armed forces.

*“As a result of the current crisis, patients are experiencing acute psychological distress. I can recall several occasions when patients could not make their appointments due to pervasive feelings of distress, fear or anxiety or a lack of family support with transportation to Armenia. Once, a patient called to reschedule their appointment because their son joined the armed forces against Azerbaijani troops, and they were stressed about what the future holds for them”. (Clinical resident, woman)*

### Affordability of cancer services

For many cancer patients, the timely initiation of comprehensive cancer treatment was affected due to affordability issues. Health professionals talked positively about policies of free anti-cancer medication provision, decentralised cancer medication supply, and co-payments for treatment implemented by the Ministry of Health (MoH) of Artsakh to support cancer patients’ treatment in Armenia. However, after the Azerbaijani military occupation, many of the social protection policies including the supply of anti-cancer medications and co-payments for treatment were posed. This meant that patients were in limbo, awaiting conflict resolution between Azerbaijan and Armenia to undergo life-savingtherapies. One health professional described substantial treatment delays in patients who could not afford cancer treatment without targeted governmental support:

*“I have patients who relocated to Armenia to start their treatment, but because of the blockade [the Lachin corridor] they cannot benefit from the financial support the Government of Artsakh used to provide for these patients. Things are different now; we cannot provide comprehensive cancer treatment to patients from Artsakh without this external funding.” [Oncologist, women].*

This was echoed in patient interviews. Patients spoke at length about financial barriers as profoundly challenging factors of their treatment. Out-of-pocket expenses linked to treatment, purchasing of anti-cancer medication, transportation and accommodation costs while undergoing treatment in Armenia were causing significant economic hardship. Despite Nagorno-Karabakh’s Government support with some anti-cancer medicines, many cancer patients had to make co-payments for radiation therapy and surgical treatments. This was distressing for patients, especially in the wake of the cost-of-living crisis and a lack of employment opportunities:

*“I am spending all your money on cancer medication and it is causing economic hardship for me and my family, especially now when there are no jobs to uptake. I’m not sure if you’re aware of this but in Artsakh, everything has doubled or tripled in price, everything is more expensive now. I am spending all my money on healthcare bills.” [Cancer patient, male]*

Two patients spoke about fake anti-cancer drugs circulating the Armenian market and the financial expenses linked to ensuring that cancer drugs they receive were produced and/or imported from abroad:

*“*There is no certainty whether you are receiving authentic or fake drugs*. *This is the reason I pay a large amount of money to receive anti-cancer medicines produced in Germany*. *I believe there is more control over the quality up there*. *This is so*, *so distressing…*. *cancer patients are suffering so much because of their illness*, *and they should also worry if medication is legitimate*.*” (Cancer patient*, *woman)**

One patient called on the Government of Nagorno-Karabakh to ensure better social protection and targeted psychosocial support for cancer patients:

*“The Government of Artsakh should take greater responsibility for protecting and supporting cancer patients, so we do not get stressed and upset over our financial bills because being diagnosed with cancer is a traumatic experience in its own right.” (Cancer patient, woman)*

## Discussion

Our findings suggest that cancer patients from former Nagorno-Karabakh faced multiple barriers in accessing cancer care services, negatively impacting their health and well-being. The Lachin corridor blockaded by Azerbaijani military troops exacerbated existing issues of approachability to cancer care services, with patients relying on the International Red Cross for medical evacuation support. The process of securing a permit and a safe transfer through a military checkpoint was described as stressful, complex, and unpredictable, risking timely access to life-saving treatments. This is against the backdrop of evidence suggesting that delay in cancer treatment by even one month is linked to a 6–13% increased risk of mortality [18]. Some patients relocated temporarily to Armenia to ensure timely treatment access to trusted services and cancer care professionals, avoiding checkpoint-associated fears and anxiety. Family support during travel and treatment was critical but not always available, causing additional distress. Out-of-pocket expenses for treatment, medication, travel, and temporary accommodation placed a significant financial burden on cancer patients, exacerbated by the humanitarian crisis. These findings align with the broader challenges faced by cancer patients seeking healthcare in conflict-affected contexts, where access is often compromised due to a multitude of factors, including disrupted healthcare systems, restricted movement, security concerns, economic instability, displacement, and the breakdown of social support networks [4,6].

Barriers to cancer care and psychosocial support services, physical and financial hardship endured by cancer patients in our study and other studies from similar contexts [7,19–22] are concerning. Cancer treatment requires a multimodal approach, starting with timely diagnosis and person-centred treatment, yet our study and previous studies report many obstacles faced by cancer patients along the continuum of cancer care pathways. This is a recurring problem in conflict-affected contexts, where healthcare infrastructure and supply chains are often compromised [4,6,7,19–23]. For instance, a recent study found that Palestinian patients with advanced-stage cancer in the West Bank experienced referral delays in accessing treatment as well as going through unpredictable, lengthy processes of obtaining Israeli permits to access cancer care in East Jerusalem [19]. The refusal or delay of Israeli permits contributed to delays in treatment, affecting patient health outcomes. Another study found that staff shortages and a lack of anti-cancer medication and diagnostic technology negatively impacted cancer patients’ access to screening and cancer management in Syria [21]. A recent retrospective study of Afghan cancer patients who had opted to receive treatment in Pakistan due to the ongoing armed conflict in Afghanistan found that only a third of adult patients received the full course of cancer treatment [22]. The contributing factors to this low completion rate were the length and safety concerns of the physical journey to reaching cancer services, as well as the need to travel with families or relatives, causing disruptions to family lives and livelihoods.

Armed conflicts and associated prolonged military occupations and/or blockades are adversities which have cumulative traumatic effects on vulnerable cancer patients, their families and communities [10]. A recent study projecting the impacts of the war in Ukraine on cancer patients argues that armed conflicts increase the risk of both developing cancer and other health-related comorbidities and complications, divert resources away from cancer care, and lead to delays in diagnosis due to competing priorities [7]. Evidence from multiple sources suggests that behavioural changes, delays to presentation, the availability of timely and affordable complex care, lack of access to facilities and infrastructure, shortage of essential medicines and medical equipment, financial hardship, loss of family structure and support, war-related stress and uncertainty about future are the key obstacles faced by cancer patients in conflict-affected regions [6, 7,19–23]. There is an urgent need to mobilise humanitarian organisations and governments across the world to ensure cancer patients and their households’ safety and facilitate rapid access to care and support services. Although guidance on comprehensive cancer care in vulnerable settings exists [24], there is still a lack of structured guidance on essential packages of cancer care in humanitarian settings and on how to assist host countries in supporting displaced communities affected by cancer [10]. More research is needed to better understand and inform cancer policies and planning in the context of armed conflicts and military blockades and how neighbouring or host countries can absorb and strategically respond to cancer care of displaced populations with cumulative traumatic experiences, such as cancer patients fleeing conflicts.

### Strengths and limitations

The strength of our study is the ability to reach cancer patients living under military blockade and amplify the lived experiences of vulnerable patients living in conflict-affected regions. We had a reasonable sample size of 24 participants, including perspectives from cancer patients and cancer care providers. We acknowledge the presence of several limitations. Firstly, the interviews with cancer patients were conducted via video calls, which may have contributed to response time limitation and missing some of the patients’ non-verbal clues. Secondly, although we were committed to sample for heterogeneity, most of the participants who agreed to take part were female. Future research should take a more targeted approach to recruitment to ensure a diverse representation of lived experiences. The sample size was relatively small; therefore, our results may have limited transferability to other conflict-affected contexts. Finally, following our interviews, on 19 September 2023, the Azerbaijani military forces occupied Stepanakert (the capital of the Nagorno-Karabakh Republic), causing mass displacement of ethnic Armenians. We lack any reliable data on how this impacted cancer patients and their access to health services in Armenia.

## Conclusion

Conflict-affected regions blockaded by military forces lack the capacity and targeted support to sustain their basic health services and provide care to those in need of life-saving treatments. Coordinated action from national and international organisations and governments is urgently needed to enhance humanitarian assistance and healthcare support to patients, their families and wider communities affected by military blockades and armed conflicts. The targets of support should be placed on: (1) Strengthening healthcare infrastructure and supply chains through establishing secure corridors for the delivery of medical supplies and prioritising the protection of healthcare infrastructure; (2) Facilitating safe passage and transportation of cancer patients by involving all parties partaking in the conflict to ensure the safety of those seeking healthcare services; (3) Strengthening collaboration and coordination with international humanitarian organisations to ensure the provision of essential healthcare services, medical evacuations, and access to care in areas where local systems are disrupted; (4) Providing mental health and psychosocial support to patients and their families; (5) Implementing financial assistance programs or subsidies to alleviate the economic burden faced by patients and their families and ensuring they can afford necessary treatments, medications, and associated costs without experiencing financial hardship; and (6) Strengthen community-based healthcare such as mobile clinics, community health workers, and telemedicine services to bridge gaps in access to care and provide essential services closer to communities affected by conflicts.

## Supporting information

S1 Checklist (DOCX)

S1 Table Interview topic guide–patients. (DOCX)

S2 Table Interview topic guide–healthcare professionals. (DOCX)
