# Supplementary material for: Barriers to access to cancer care for patients from the conflict-affected region of the Nagorno-Karabakh Republic: A qualitative study
Source: PLOS Glob Public Health. 2024 Jul 1;4(7):e0003243. doi: 10.1371/journal.pgph.0003243 (PMC11216571; doi:10.1371/journal.pgph.0003243)
Supplement: S1 Table — (DOCX) [file pgph.0003243.s002.docx]

# **S1 Table. Interview topic guide – Patients**

| - Which hospital do you usually go to see a cancer specialist? [ask about geographical location, frequency of seeking care from there, and why they usually go there]. - How do you get to the hospital for your appointments locally in NKR/in Yerevan? [ ask if using a car, bus, taxi]. - Do you have to move away from family when you need to go to a hospital to see a cancer specialist or to get cancer treatment? - Why do you have to move away, and how long are you usually away from your home? - Where do you stay when you need to go to the hospital to see a cancer specialist or to get cancer treatment in Yerevan? - Do you have someone coming with you for your cancer appointments and cancer treatments? [If yes, who?] - What difficulties do you currently face when getting to your cancer appointments? - What difficulties do you currently face with cancer treatment? Is the treatment generally affordable for you? - Are there things you would like to tell me regarding the availability and accessibility of cancer specialists/ hospitals in your hometown and Yerevan? - How would you like cancer services to be improved for patients like you? How can policy-makers make these changes sustainable? |
| --- |
