# Supplementary material for: Barriers to access to cancer care for patients from the conflict-affected region of the Nagorno-Karabakh Republic: A qualitative study
Source: PLOS Glob Public Health. 2024 Jul 1;4(7):e0003243. doi: 10.1371/journal.pgph.0003243 (PMC11216571; doi:10.1371/journal.pgph.0003243)
Supplement: S2 Table — (DOCX) [file pgph.0003243.s003.docx]

# **S2 Table. Interview topic guide – Healthcare professionals**

| - Can you tell me about your experiences with providing cancer care to patients from NKR? - What do you see as the main issues facing cancer patients from NKR? - Do you think that the cancer experiences of people from NKR are different in any way from the experiences of local patients? If so, in what way? - Are there any special considerations when treating cancer patients from NKR? - What type of things do cancer patients from NKR find most challenging about their cancer treatments? - Are there any difficulties with completing treatment? Are these different from local patients? - Are you aware of any cultural issues that come up for patients needing these treatments? - Could you describe the key issues that cancer patients from NKR patients need support with? - What types of support services are there for these patients? Do you know if they use these supports? - Are there other support services that you feel would be beneficial for cancer patients from NKR? - Do you refer cancer patients to any support services? If so, which services you would refer to? - How would you like cancer services to be improved for patients from NKR? How can policy-makers make these changes sustainable? - Is there anything you would like to add in relation to what we have discussed so far? |
| --- |
